# Prdx1 promotes the loss of primary cilia in esophageal squamous cell carcinoma

**DOI:** 10.1186/s12885-020-06898-y

**Published:** 2020-05-01

**Authors:** Qiongzhen Chen, Jinmeng Li, Xiaoning Yang, Junfeng Ma, Fanghua Gong, Yu Liu

**Affiliations:** 1 College of Life and Environmental Science, Wenzhou University, Wenzhou, China; 2 School of Pharmacy, Wenzhou Medical University, Wenzhou, China; 3 The first affiliated hospital of Wenzhou Medical University, Wenzhou, China

**Keywords:** Prdx1, Cilia, ESCC, Invasion, Tumorigenesis

## Abstract

**Background:**

Loss of primary cilia is frequently observed in tumor cells, suggesting that the absence of this organelle may promote tumorigenesis through aberrant signal transduction, the inability to exit the cell cycle, and promotion of tumor cell invasion. Primary cilia loss also occurs in esophageal squamous cell carcinoma (ESCC) cells, but the molecular mechanisms that explain how ESCC cells lose primary cilia remain poorly understood.

**Methods:**

Inhibiting the expression of Prdx1 in the ESCC cells to detect the up-regulated genes related to cilium regeneration and down-regulated genes related to cilium disassembly by Gene chip. And, mice and cell experiments were carried to confirm the role of the HEF1-Aurora A-HDAC6 signaling axis in ESCC.

**Results:**

In this study, we found that silencing Peroxiredoxin 1 (Prdx1) restores primary cilia formation, and over-expressing Prdx1 induces primary cilia loss in ESCC cells. We also showed that the expression of Prdx1 regulates the action of the HEF1-Aurora A-HDAC6 signaling axis to promote the disassembly of primary cilia, and suppression of Prdx1 results in decreased tumor formation and tumor mass volume in vivo.

**Conclusions:**

These results suggest that Prdx1 is a novel regulator of primary cilia formation in ESCC cells.

## Background

The primary cilium is an antenna-like organelle present on the surface of most types of mammals, with an extracellular signal transduction function that regulates cell growth and development. Cilium disassembly is closely associated with cell cycle progression and external signal transduction [1–3]. Cilium disassembly occurs when cells proliferate and then restoration occurs once mitosis is complete [4]. Structural or functional cilia anomalies are associated with many genetic diseases, which are collectively called ciliopathies [5, 6]. Recent studies have shown that cilia are closely associated with tumorigenesis [7–9], and primary cilia are disassembled or lost in many tumor tissues, including esophageal squamous carcinoma (ESCC) [3, 10], colon cancer [11], breast cancer [12], hepatic carcinoma [13], cholangiocarcinoma [14], and thyroid cancer [15]. This suggests that inhibition of cilia disassembly or loss in tumor cells or the promotion of ciliary regeneration may inhibit the proliferation and invasion of tumor cells.

ESCC is the major histological subtype of esophageal carcinoma. Recent studies have shown that 456,000 people are affected by esophageal carcinoma each year, of which esophageal squamous carcinoma accounts for about 90% [16, 17]. ESCC is one of the most aggressive tumors worldwide, with a five-year survival rate that is less than 20% [18, 19]. Prdx1 is an antioxidant protein that is widely expressed in eukaryotic cells, protects cells from ROS damage [20], and regulates cell signal transduction by controlling cell proliferation and H_2_O_2_ activity. The abnormal expression of Prdx1 has been reported for many kinds of tumors [21, 22]. Several studies have shown that Prdx1 is highly expressed in ESCC, and Prdx1 is closely related to the disassembly of primary cilia in tumors [23]. Thus, Prdx1 and primary cilia may play an important role in the development of ESCC, making Prdx1 an exciting potential target for tumor treatment. However, the detailed molecular mechanism by which Prdx1 regulates cilia disassembly in ESCC remains unclear.

Our previous research demonstrated that the evolutionary conserved Prdx1 participates in the regulation of cilium disassembly, and decreases the activity of Aurora A, a marker protein of cilium disaggregation [3, 10]. In this study, we expanded that work and found that inhibiting the expression of Prdx1 in the ESCC cells significantly up-regulated genes related to cilium regeneration and down-regulated genes related to cilium disassembly. Cell and animal experiments were performed and demonstrated that Prdx1 plays a regulatory role in the key signal axis of cilium disassembly, NEDD9 (HEF1)-Aurora A-HDAC6, and affects the tumorigenicity and invasiveness of ESCC cells. Overall, our results indicate that Prdx1 contributes to disassembly of primary cilia by controlling the NEDD9-Aurora A-HDAC6 signal axis in ESCC.

## Methods

### Cell strains and animals

The human esophageal squamous carcinoma cell line EC9706 was obtained from the State Key Laboratory of Molecular Oncology, Chinese Academy of Medical Sciences. The experimental cells were divided into seven groups, normal EC9706 cells, EC9706 cells transfected with shPrdx1 lentivirus (shPrdx1), EC9706 cells transfected with negative control lentivirus (shControl), EC9706 cells transfected with OE-Prdx1 lentivirus (OE-Prdx1), EC9706 cells transfected with corresponding negative control lentivirus (OE-Control), EC9706 cells treated with Tripolin A (EC9706 + Tripolin A), and EC9706 cells transfected with OE-Prdx1 lentivirus and treated with Tripolin A (OE-Prdx1 + Tripolin A). Healthy male BALB/c nude mice (4 weeks, *n* = 20, 10 in each group) were provided by the Animal Center of the Chinese Academy of Science (Shanghai, China). All animals were raised and treated according to guidelines developed by the National Institutes of Health guide for the care and use of laboratory animals (NIH Publications No. 8023, revised 1978). The mice were housed under strictly controlled SPF environmental conditions and mice were sacrificed by CO_2_ inhalation.

### Acquisition of lentivirus

The Prdx1 interfering and negative control lentivirus plasmid were constructed by inserting the target sequence (shPrdx1: TGTCTGACTACAAAGGAAA, shControl: TTCTCCGAACGTGTCACGT) into the Age I/EcoR I restriction enzyme cutting sites of the GV112-Lentivirus vector according to previous results [3]. The over-express Prdx1 (Accession Number: NM_001202431) and control plasmid were constructed by inserting the target sequence into the Age I/BamHI restriction enzyme cutting sites of the GV287-Lentivirus vector. Plasmid vectors were obtained from the Genechem Co. Ltd. (Shanghai, China). The synthesis of the target gene sequence, establishment of the plasmid carrier, and lentivirus packaging were performed as described previously [3].

### Cell culture and lentivirus transfection

The EC9706 cells were cultured in RPMI-1640 media (Gibco) containing 10% fetal bovine serum (FBS, Gibco) in a humidified atmosphere of 5% CO_2_ at 37 °C. The EC9706 cells (MOI = 100) were infected with shPrdx1 lentivirus (1 × 10^9^ TU/ml) or the over-expressed Prdx1 lentivirus (1 × 10^8^ TU/ml) to decrease or increase the levels of Prdx1. The corresponding negative control lentivirus was similarly transfected into cells. The procedure was as follows. EC9706 cells were inoculated into a 6-well microplate at a density of 1 × 10^5^/ well. Next, 1 ml of culture medium without antibiotics was added to each well when the cells grew to 30% confluence. Then, 5 mg/ml polybrene and the lentivirus were added and cultured for 12 h at 37 °C, followed by the addition of 2 ml of fresh culture medium. The expression of GFP fluorescent protein was measured as an indicator of the efficiency of lentivirus transfection using an inverted fluorescence microscope. The cells were collected at 72 h for further experiments.

### Gene chip detection

Gene chip detection was used to detect the changes in gene and signal pathways in ESCC cells following Prdx1 interference. The total RNA of control EC9706 cells and cells in which Prdx1 was inhibited were isolated using the Trizol reagent. The extracted total RNA samples were subjected to quality testing with a NanoDrop 2000 and an Agilent Bioanalyzer 2100. The qualified samples were then applied to a human gene expression profile chip (chip type: GeneChip primeview human, number: 901838) developed by Affymetrix. The hybridization, washing of the gene chip, scanning, and data analysis were completed by the Genechem Co. Ltd. (Shanghai, China).

### Tumor formation in nude mice

Negative control EC9706 cells in logarithmic growth phase and EC9706 cells with inhibited levels of Prdx1 were prepared as cell suspensions and injected subcutaneously into the right axillary of nude mice (2 × 10^7^ /ml, 200 μl for each). The body weight and tumor volume of each nude mouse were measured twice each week. Each nude mouse was sacrificed under anesthesia at 23 d and the mice were put into a live imaging device (IVIS® Lumina III) for luciferase luminescence detection, and the tumor was removed, weighed, photographed, and preserved after the mouse was sacrificed.

### Immunofluorescence detection of cell cilia

In order to induce cell cilia formation, the cells were inoculated into a 24-well plate. When the cell density was 80%, the culture medium was replaced by fresh serum-free medium for 4–6 d. To measure immunofluorescence, slides with cells were fixed with 4% polyformaldehyde for 30 min, incubated with glycine (2 mg/ml) for 5 min, and then blocked with 5% BSA for 20 min. The rabbit anti-acetyl-α-Tubulin (1:500, Abcam) antibody was then added and incubated overnight at 4 °C. After washing with PBST three times, the cells were then incubated with sheep anti-rabbit antibody (1:200, Santa Cruz), labeled with IgG/TR for 1 h at 37 °C, and then washed with PBST three times. Nucleus staining was performed by DAPI reagent for 7 min and then the cover slide was sealed. Positive fluorescence microscopy (ECLIPSE NI, Nikon, Japan) was used to observe and photograph cells.

### Quantitative real-time PCR

cDNA was synthesized from total RNA (1 μg) purifified using TRIzol reagent, and then quantitative real-time reverse transcription PCR (qRT-PCR) was performed on a Rotor-Gene 6000 thermocycler (Corbett Research, Sydney, Australia) using a KAPA SYBR FAST Universal 2X qRT-PCR Master Mix (Kapa Biosystems, Woburn, MA, USA). The primers for FGFR1, IGF1R, ABI2 and NEDD9, Aurora A, HDAC6 were designed by Sangon Biotech (Shanghai, China). PCR reaction parameters were as follows: 1 cycle of 95 °C for 3 min, followed by 40 cycles each comprising three steps of 95 °C for 3 s, 55 °C for 15 s, and 72 °C for 30 s. All PCR samples were prepared in triplicate and the relative mRNA expression levels were normalized to GAPDH and determined by the 2^−ΔΔCt^ method.

### Transwell invasion experiment

Transwell invasion chambers (article number: 354480, Corning) were used to detect cell invasion capacity. Serum-free medium was preheated at 37 °C, added to the upper chamber, and incubated in a 37 °C incubator for 2 h. After discarding the medium, 500 μl fresh medium containing 10% FBS was added to the lower chamber and 300 μl cell suspension (2 × 10^5^ cells/ml) was added to the upper chamber. After incubation for 24 h, the chamber was removed, washed twice with PBS, and then fixed with 5% glutaraldehyde for 30 min at 4 °C. The cells were stained with 1% crystal violet staining solution for 20 min and then washed three times with PBS. The cells on the upper surface were cleaned with cotton balls, and we photographed the cells in the under surface of the chamber using an inverted microscope (Nikon, Japan). The mean value was determined by averaging the number of cells counted in nine visual fields.

### Western-blot experiment

Western-blotting was used to detect and analyze the levels of proteins in cells and tumor tissues. Total proteins were extracted from cells or tissues, quantified with the BCA reagent method, and then separated by 8% or 10% (w/v) SDS-PAGE. The protein was transferred to a PVDF membrane (Bio-Rad, Hercules), blocked for 1.5 h with 5% (w/v) skim milk in TBST at room temperature, and incubated overnight at 4 °C with the Prdx1 antibody (1:10000, Abcam), NEDD9 antibody (1:1000, Cell signaling technology), p-Aurora A antibody (1:1000, Gentex), Aurora A antibody (1:10000, Abcam), HDAC6 antibody (1:10000, Abcam), or the GAPDH antibody (1:10000, Abcam). The membranes were washed three times with TBST and then incubated with horseradish peroxidase-conjugated (HRP) secondary antibody for 1 h at room temperature. The protein signal was detected using a protein gel imaging system (ChemiDocXRS + Imaging System; Bio-Rad).

### Statistical analysis

All data were expressed as the mean ± SEM. A one-way analysis of variance and t test were performed to compare the differences among various samples. All data were analyzed using the software GraphPad Prism 5.0. The difference was statistically significant if *P* < 0.05.

## Results

### Inhibition of prdx1 restored primary cilia and suppressed tumor invasion

In order to study the effect of inhibition of Prdx1 on cilia and cell function, we used gene chip detection followed by experiments in cells to verify the findings. A lentivirus (shPrdx1) was transfected into EC9706 cells to inhibit the expression of Prdx1. EC9706 cells transfected with shPrdx1 lentivirus and shControl lentivirus (negative control) were subjected to gene chip analysis to study the potential changes in gene expression and signal pathways of cells after inhibition of Prdx1 .

**Fig. 1:**
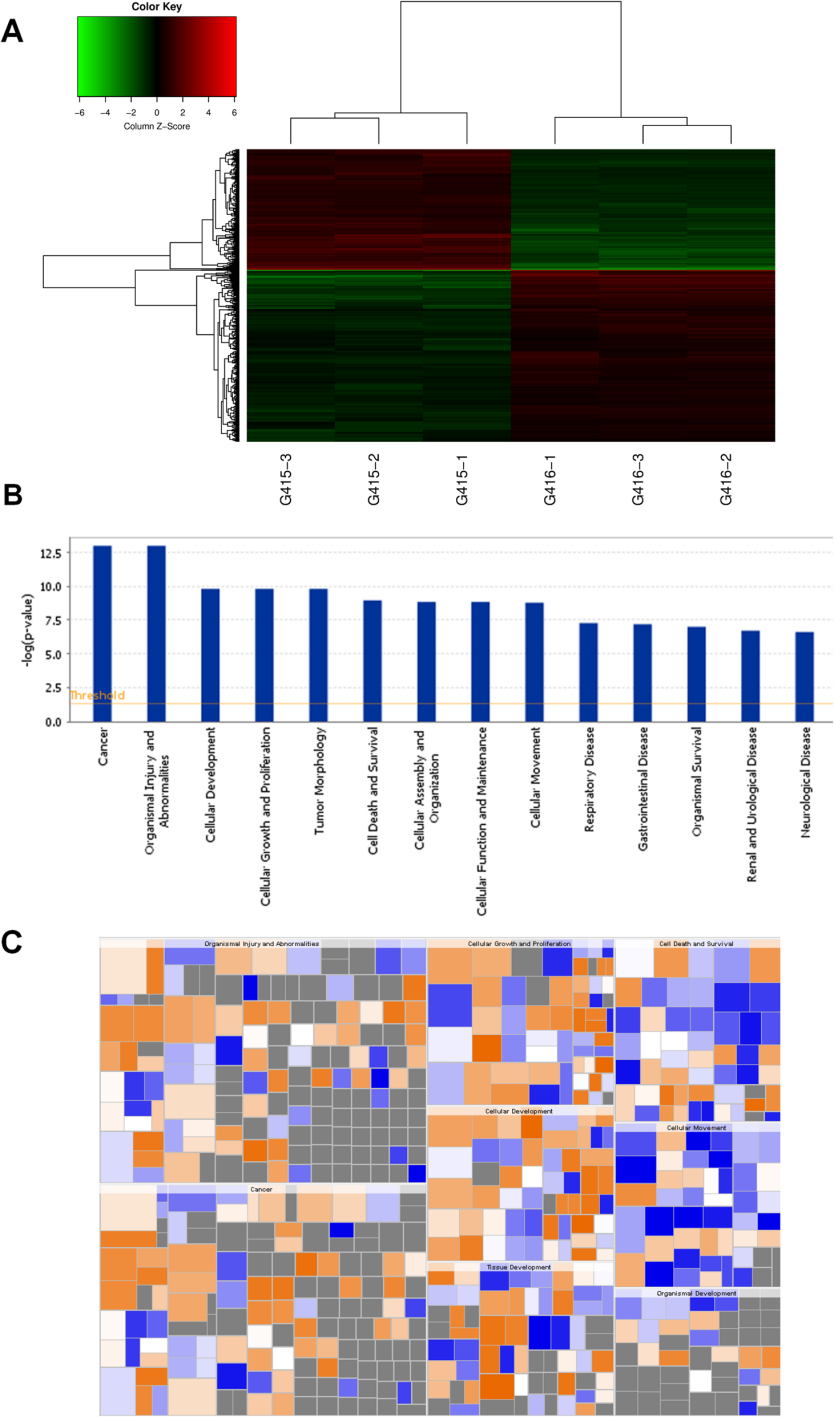
Gene chip detection after Prdx1 inhibition. **a** Changes of relevant genes in cells after inhibition of Prdx1. Negative control group: G415–1, G415–2, and G415–3. shPrdx1 group: G416–1, G416–2, and G416–3. The screening standard for a significant difference in gene expression was a value of |FC| greater than 1.3 and a *P*-value less than 0.05. Red signifies that the signal value of the gene is relatively up-regulated. Green signifies that the signal value of the gene is relatively down-regulated. Black signifies a moderate signal value of the gene. Grey signifies that no signal value was detected. **b** Differentially expressed genes in disease and cell functional classifications after Prdx1 inhibition in EC9706 cells. All diseases and cell functions were sequenced with -Log (*P*-value). **c** The effects of differentially expressed genes on cell function and diseases after Prdx1 inhibition. Orange signifies Z-score > 0. Blue signifies Z-score < 0. Grey signifies no Z-score value. Z-score > 2 signifies that the function is significantly activated. Z-score < − 2 signifies that the function is significantly inhibited

The gene chip detection results showed that the Prdx1 gene level decreased (FC: − 5.9895, *P*-value: 1.04 × 10^− 7^) significantly in EC9706 cells transfected with the shPrdx1 lentivirus. There were 512 up-regulated genes and 340 down-regulated genes relative to the negative control group when Prdx1 was inhibited (Fig. 1a and Table S1), using a screening standard specifying a significant difference in gene expression as a value of |FC| greater than 2 and a *P*-value less than 0.05. We studied the relationship between the up-regulation and down-regulation of differentially expressed genes with the activation or inhibition of diseases and cell functions (Fig. 1b, c). Genes related to the formation of cellular protrusions (cilia) were significantly activated, and genes influencing the invasion of tumor cells (cell movement of the carcinoma cell line) were significantly inhibited after the inhibition of Prdx1 (Table 1). The enrichment of differentially expressed genes in disease and cell functional classifications are presented in Table 1 and Table S2. We also studied the occurrence of significantly differentially expressed genes in the classical signaling pathway based on the public database and the results of gene detection, and the results are presented in Table S3 and Fig. S1. In order to confirm the gene chip results, we used RT-PCR, and the results showed that the mRNA level of FGFR1, IGFR1, and ABI2 were significantly increased after the inhibition of Prdx1 (Fig. S2A-D), and the mRNA level of NEDD9, Aurora A and HDAC6 were significantly decreased after the inhibition of Prdx1 (Fig. S2E-G). Studies have shown that FGFR1 knockout can cause ciliary shortening [24, 25], IGFR1 plays an important role in ciliary elongation [26, 27], and ABI2 is a tumor suppressor and a cell migration suppressor, participating in human head and neck squamous cell carcinoma [28]. And NEDD9 and Aurora A was also known as important oncogenes regulating, correspondingly, cell division and cell invasion [29–32]. The changes in the mRNA levels of these genes suggest that Prdx1 may play an important role in the formation of cilia and the invasion of ESCC.

**Table 1:** Effects of differentially expressed genes on disease and cell function after Prdx1 inhibition

Diseases or Functions Annotation	Activation z-score	***p***-Value	Differentially expressed genes (n)
Tumorigenesis of tissue	−0.137	4.02E-13	656
Proliferation of tumor cells	0.927	1.52E-10	56
Proliferation of cells	−0.89	5.22E-10	269
Migration of tumor cell lines	−0.848	1.62E-09	76
Cell proliferation of tumor cell lines	−0.281	7.1E-09	134
Microtubule dynamics	1.768	1.43E-08	106
Apoptosis of tumor cell lines	1.059	1.62E-08	108
Invasion of cells	−0.289	5.28E-08	82
Cell death of tumor cell lines	1.059	5.87E-08	127
Formation of cellular protrusions	2.545	0.00000043	82
Cell movement of carcinoma cell lines	−2.11	0.00000964	25
Migration of carcinoma cell lines	−2.082	0.0000125	22
Invasion of tumor cell lines	−0.362	0.000000636	64
Attachment of cells	2.187	0.000382	15
Engulfment of tumor cell lines	−1.75	0.000264	16
Gastrointestinal tract cancer	−0.926	0.000329	491
Extension of neurites	1.946	0.000335	18
Migration of pancreatic cancer cell lines	−1.912	0.000338	8
Development of kidney cell lines	−1.118	0.000369	7
Cell proliferation of kidney cell lines	−1.154	0.000426	18
Colony formation of lung cancer cell lines	−1.153	0.000448	8
Invasion of digestive organ tumor	−1	0.000512	8
Invasion of epithelial cell lines	−1.577	0.000598	7
Metastatic potential of tumor cell lines	−1.404	0.000738	6
Extension of cellular protrusions	1.257	0.00075	20
Apoptosis of colorectal cancer cell lines	1.064	0.000782	22
Degeneration of nervous system	−2.101	0.000892	26
Migration of prostate cancer cell lines	−1.087	0.000924	12
Degeneration of neurons	−1.153	0.00133	22
Neurodegeneration	−1.99	0.00136	27

Z-score > 2 signifies that the function is significantly activated. Z-score < −2 signifies that the function is significantly inhibited. All diseases and functions are sorted using -Log (*P*-value), ranking from high to the bottom according to the value of -log (*p*-value)

**Fig. 2:**
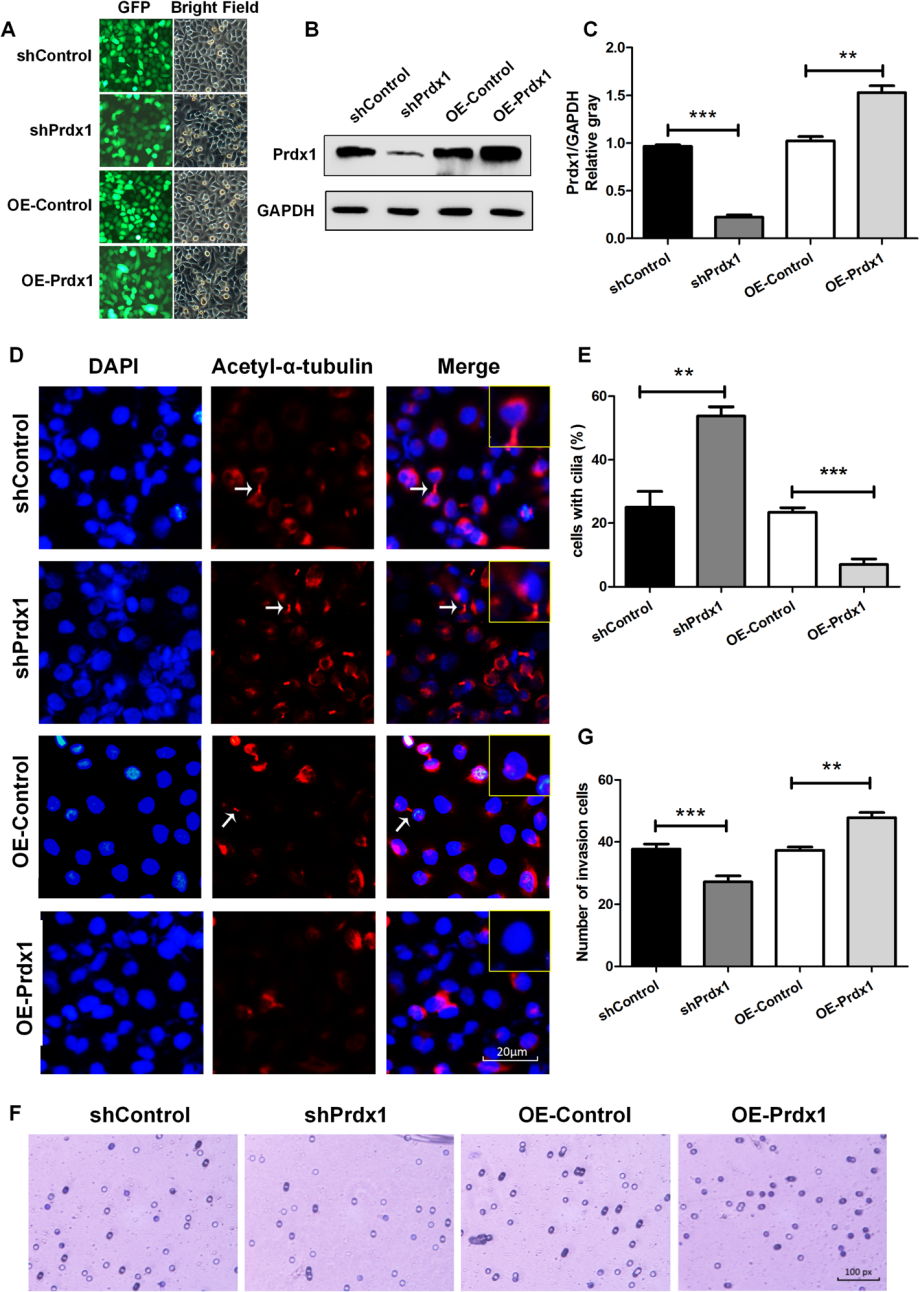
Inhibition of Prdx1 restored primary cilia and suppressed tumor invasion. **a** Transfection efficiency of lentivirus in EC9706 cells. EC9706 cells were transfected with plasmids expressing shRNA or OE-RNA and green fluorescent protein. Green fluorescent protein was detected as a marker of transfection efficiency by fluorescence microscopy (100×). **b** Western blotting was used to analyze the expression of Prdx1 in each group. GAPDH served as a loading control. Full-length blots/gels are presented in Supplementary Fig. X. **c** Analysis of western blot signals using Image J. The relative protein levels are indicated as bars. All experiments were performed in triplicate and results are expressed as mean ± SEM. **d** Immunofluorescence was used to detect the changes of the cilium marker protein (red: acetyl-α-tubulin, blue: DAPI, white arrows: cilia). **e** Number of cells with cilia (%) in each group. **f** Transwell invasion experiment to detect the changes in cell invasion capacity. **G** Number of invasion cells in each group. *P < 0.05, **P < 0.01, ***P < 0.001

We also conducted a cell culture experiment to study the effect of Prdx1 on ESCC. The Prdx1 interference lentivirus (shPrdx1), the over-expressed Prdx1 (OE-Prdx1) lentivirus, and corresponding negative control lentivirus (shControl and OE-Control) were used to separately transfect EC9706 cells. The expression of Prdx1 was decreased compared with the level in the negative control group (shControl) after transfection with the shPrdx1 lentivirus, and the expression of Prdx1 was increased compared with the level in the negative control group (OE-Control) after transfection with the OE-Prdx1 lentivirus (Fig. 2a-c). To study the effect of Prdx1 on cilia, an immunofluorescence experiment was used to detect acetyl-α-tubulin, a cilium marker protein. The results showed that the amount of cells with cilia (%) was increased by more than 2-fold after Prdx1 was inhibited, and in the OE-Prdx1 lentivirus group, the amount of cells with cilia (%) was decreased by more than 3.1-fold compared with the negative control group (Fig. 2d, e). A Transwell invasion experiment was conducted to study the effect of Prdx1 on the invasion of tumor cells, and the results showed that compared with the control group, there was a 30% decrease of tumor invasion capacity after Prdx1 inhibition and a 32% increase of tumor invasion capacity after Prdx1 was over-expressed (Fig. 2f, g). The above results were consistent with the results of the gene chip detection, which indicated that the inhibition of Prdx1 in the EC9706 cells could promote cilia regeneration and inhibit the invasion capacity of the tumor cells.

### Prdx1 regulated the expression of the cilium disassembly signal axis of NEDD9-Aurora A-HDAC6 in ESCC

**Fig. 3:**
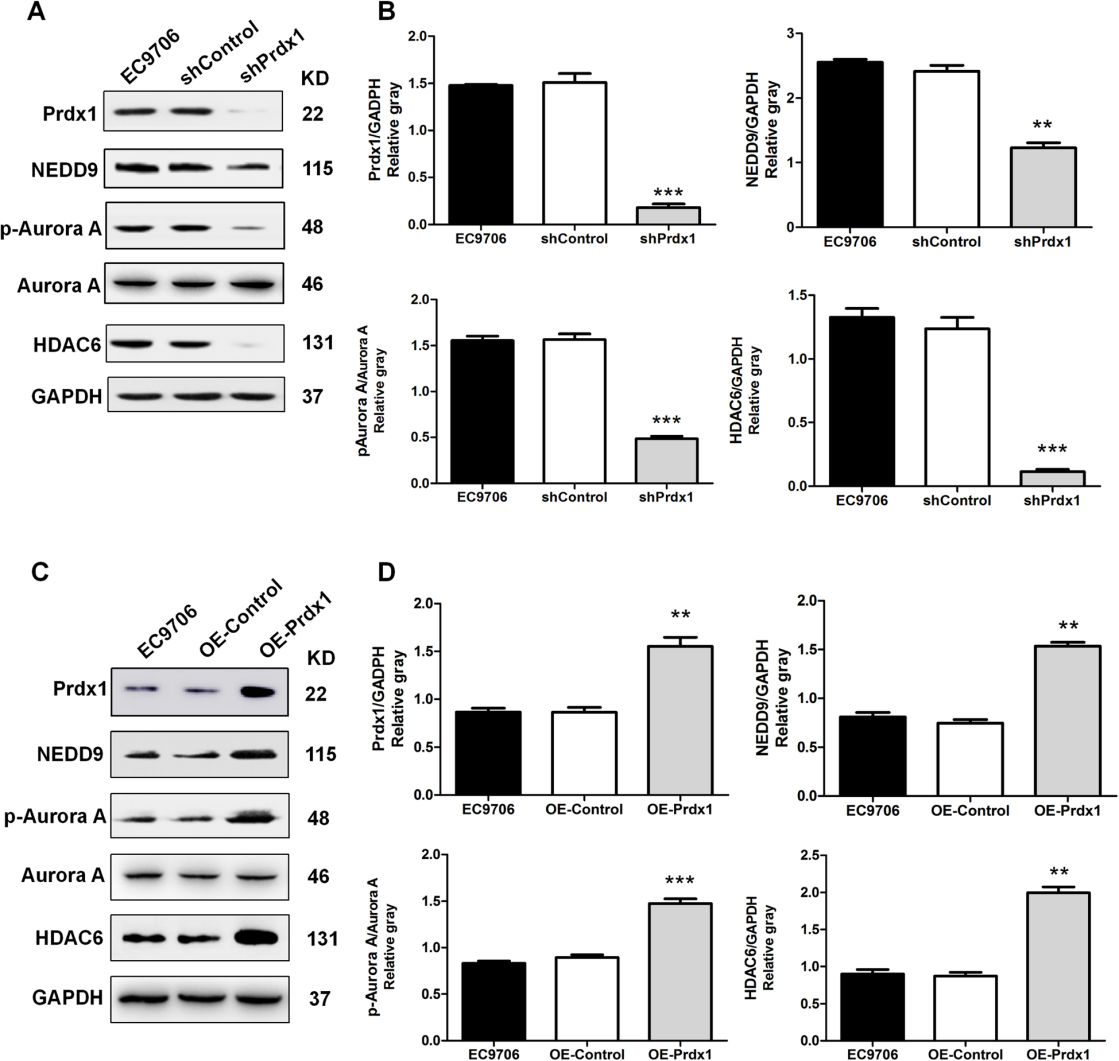
Prdx1 significantly regulated the expression of NEDD9-Aurora A-HDAC6 in EC9706 cells. **a** Western-blot was used to detect the expression levels of Prdx1, NEDD9, p-Aurora A, Aurora A, HDAC6, and GAPDH in cells of each group. **b** Analysis of western blot signals using Image J. GAPDH was used as a loading control. **c** Changes of the expression of Prdx1, NEDD9, p-Aurora A, Aurora A, HDAC6, GAPDH in cells of each group. **d** Analysis of western blot signals using Image J. GAPDH was used as a loading control. The relative protein levels are indicated as bars. Full-length blots/gels are presented in Supplementary Fig. X. All experiments were performed in triplicate and the results are expressed as mean ± SEM. *P < 0.05, **P < 0.01, ***P < 0.001

In previous work, we demonstrated that inhibition of Prdx1 in EC9706 cells could promote cilia regeneration and down-regulate the expression of the key cilium disassembly protein, Aurora A. Several studies have suggested that the scaffolding protein NEDD9 (HEF1) can activate the oncogenic Aurora A (AurA) kinase at the basal body of cilia, which in turn causes phosphorylation and activation of HDAC6, a tubulin deacetylase, promoting cilium disassembly [29–32]. HDAC6 inhibition can enhance the efficacy of anticancer agents in cancers and suppress ESCC proliferation [33, 34]. Thus, we speculated whether Prdx1 could influence the expression of the cilia disassembly signal axis NEDD9-Aurora A-HDAC6. The gene chip and RT-PCR results indicated that the mRNA levels of NEDD9, Aurora A, and HDAC6 were significantly decreased in response to inhibition of Prdx1 (Fig. S2D-F). Cells containing the shPrdx1 lentivirus or negative control (shControl) lentivirus were tested and showed that the expression levels of NEDD9, p-Aurora A, and HDAC6 were significantly decreased in Prdx1-inhibited cells (Fig. 3a, b). The OE-Prdx1 lentivirus increased the expression of Prdx1, and the levels of NEDD9, p-Aurora A, and HDAC6 were also up-regulated when Prdx1 was overexpressed (Fig. 3c, d). These results showed that Prdx1 regulated the expression of NEDD9, Aurora A, and HDAC6 in EC9706 cells, and suggest the promoting effect of Prdx1 on cilia disassembly and tumor invasion may be through regulation of the NEDD9-Aurora A-HDAC6 signal axis.

### Tripolin a reversed the effects of over-expressed Prdx1 on cilium disassembly and tumor invasion

**Fig. 4:**
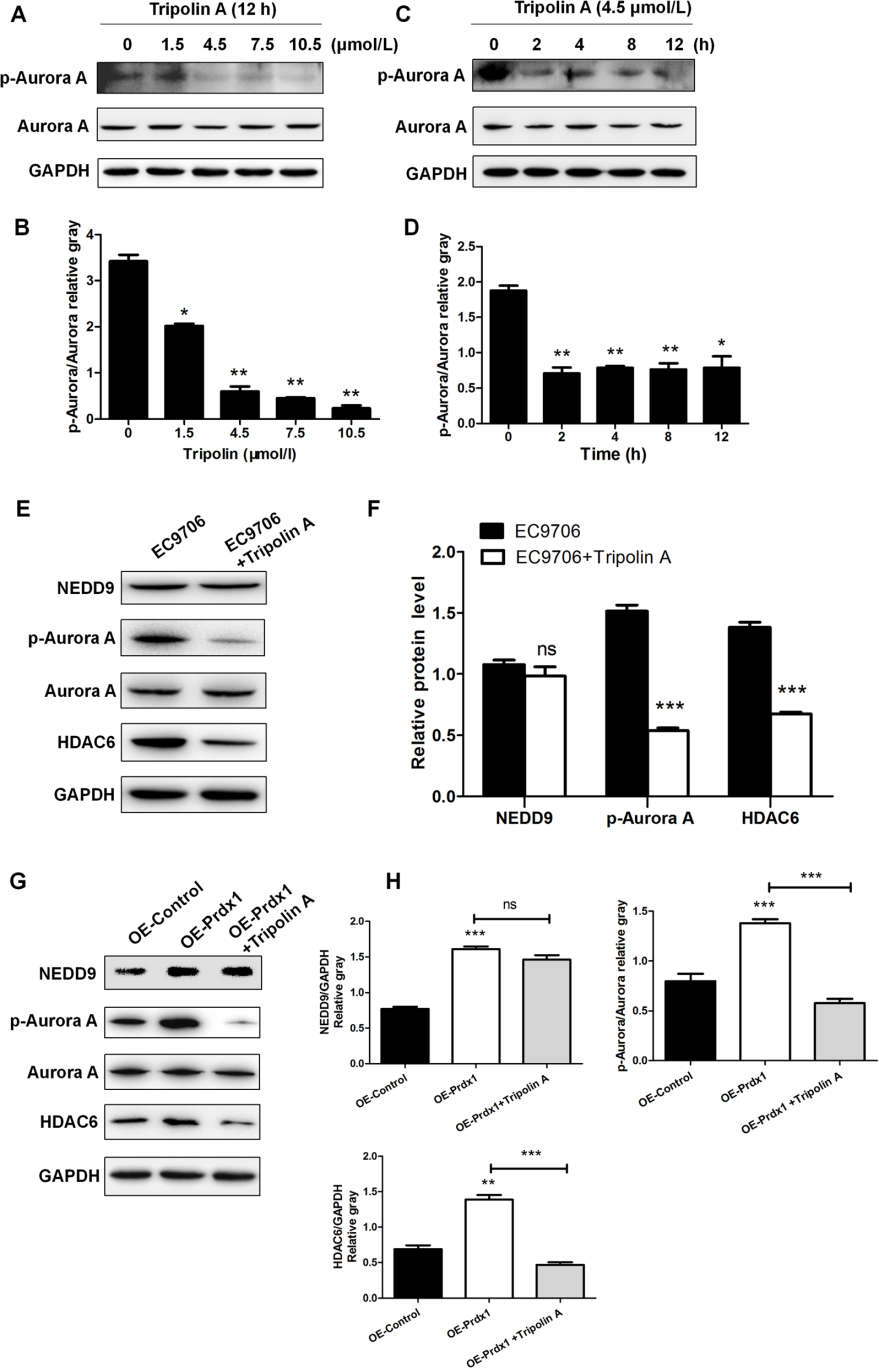
Tripolin A reversed the promotion effect of over-expressed Prdx1 on p-Aurora A and HDAC6. **a** Western-blot was used to detect changes of Aurora A expression in EC9706 cells treated with different concentrations (0, 1.5, 4.5, 7.5, and 10.5 μmol/L) of Tripolin A. **b** Relative gray level for p-Aurora A/Aurora A in cells after treatment with different concentrations of Tripolin A. **c** Changes of p-Aurora A/Aurora A expression in EC9706 cells treated with 4.5 μmol/L Tripolin A for different lengths of time (0, 2, 4, 8, and 12 h). **d** Relative gray level of p-Aurora A/Aurora A in cells after treatment with 4.5 μmol/L Tripolin A for different lengths of time. **e** The expression of NEDD9, p-Aurora A, Aurora A, and HDAC6 after EC9706 cells treated with 4.5 μmol/L Tripolin A for 2 h. **f** Analysis of western blot signals using Image J. GAPDH was used as a loading control. **g** The expression of NEDD9, p-Aurora A, Aurora A, and HDAC6 in different treatment groups. The OE-Prdx1 group was EC9706 cells transfected with the OE-Prdx1 lentivirus, and then treated with 4.5 μmol/L Tripolin A for 2 h. **h** Relative expression of NEDD9, p-Aurora A and HDAC6 in cells of different groups. GAPDH was used as a loading control. Full-length blots/gels are presented in Supplementary Fig. X. *P < 0.05, **P < 0.01, ***P < 0.001

**Fig. 5:**
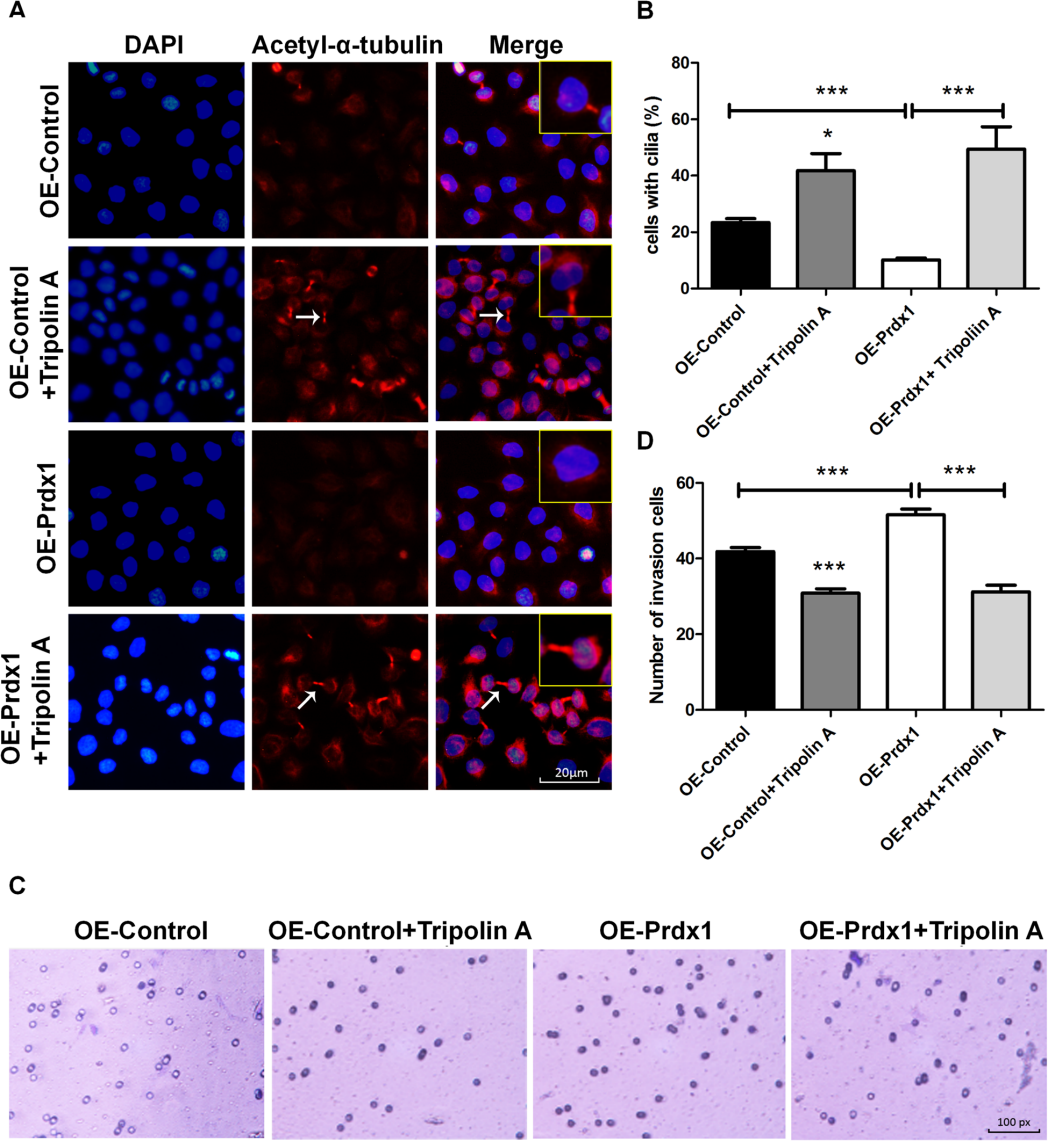
Tripolin A reversed the promotion effect of over-expressed Prdx1 on cilium disaggregation and tumor invasion. **a** Immunofluorescence was used to detect changes of the cilium marker protein (red: acetyl-α-tubulin, blue: DAPI, white arrows: cilia) in different groups. **b** Number of cells with cilia (%) in each group. **c** Transwell invasion experiment to measure cell invasion capacity in each group. **d** Number of invasion cells in each group. Data are presented as mean ± SEM, *P < 0.05, **P < 0.01, ***P < 0.001

We found that Prdx1 could regulate the expression of NEDD9, Aurora A, and HDAC6 in ESCC, but the relationship between these expression effects in ESCC and whether the action of Prdx1 to promote cilium disassembly and tumor invasion were achieved through regulation of the NEDD9-Aurora A-HDAC6 signal axis were inconclusive. Thus, we next used the Aurora A inhibitor Tripolin A to inhibit the activity of Aurora A (p-Aurora A), to investigate the relationship of Prdx1 with NEDD9-Aurora A-HDAC6 signal axis. We stimulated EC9706 cells with different concentrations of Tripolin A (0, 1.5, 4.5, 7.5, or 10.5 μmol/L) for 12 h, and observed that the expression of p-Aurora A decreased in a dose-dependent manner (Fig. 4a, b). EC9706 cells were stimulated with 4.5 μmol/L Tripolin A for 0, 2, 4, 8, and 12 h, and we observed that the expression of p-Aurora A gradually decreased (Fig. 4c, d). Based on these results, we stimulated EC9706 cells at a concentration of 4.5 μmol/L for 2 h and observed that the expression of Aurora A and HDAC6 decreased significantly (Fig. 4e, f), suggesting that Aurora A could regulate the expression of HDAC6 in ESCC. Use of 4.5 μmol/L Tripolin A to stimulate cells for 2 h inhibited the activity of Aurora A (p-Aurora A) and blocked the NEDD9-Aurora A-HDAC6 signal axis. Under these conditions, over-expressed Prdx1 no longer enhanced the expression of HDAC6 (Fig. 4g, h) and the effects of Prdx1 to promote cilia depolymerization (Fig. 5a, b) and cell invasion (Fig. 5c, d) weakened. Thus, the above results suggest that Prdx1 could promote cilia disassembly and tumor invasion in ESCC, and these promoting effects on cilium disassembly and tumor invasion were mediated through the NEDD9-Aurora A-HDAC6 signal axis.

### Inhibition of Prdx1 decreased tumorigenesis, reduced tumor volume and weight, and promoted cilium regeneration

**Fig. 6:**
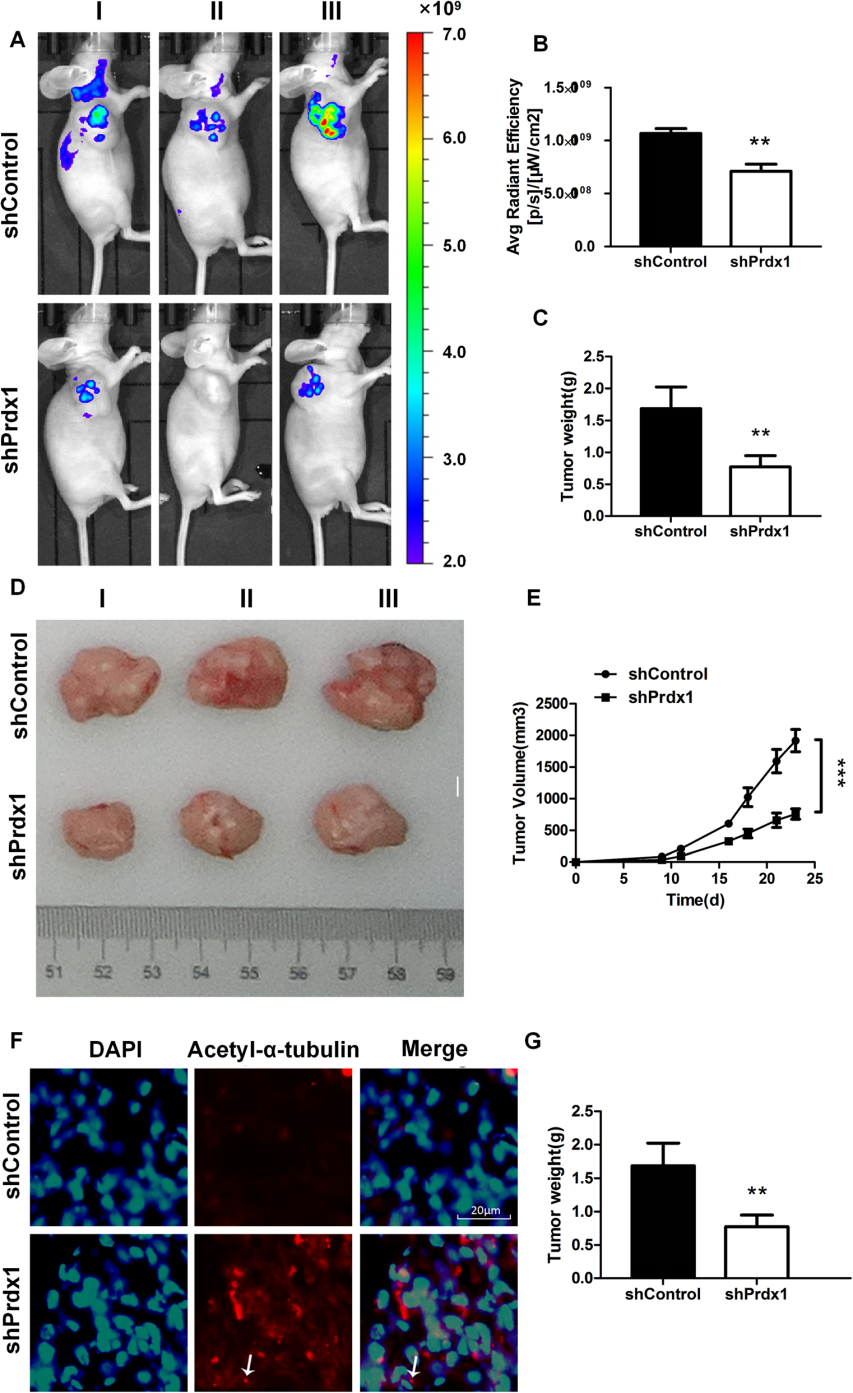
Inhibition of Prdx1 decreased tumorigenesis, reduced tumor volume and weight, and promoted cilia regeneration. **a** Live imaging was used to detect the tumorigenesis, and the radiant efficiency and distribution of luciferase luminescence were used as indicators of tumor formation ability and tumor invasive capacity, respectively. **b** The radiant efficiency of luciferase luminescence in each group. **c** Changes of tumor weight after inhibition of Prdx1. **d-e** Changes of tumor volume in each group. **f** Immunofluorescence assay to detect the cilium marker protein (red: acetyl-α-tubulin, blue: DAPI, white arrows: cilia) in tumor tissue of each group. **g** Changes in the number of cells with cilia (%) in tumor tissue of each group. *P < 0.05, **P < 0.01, ***P < 0.001

We next conducted an animal experiment to test the effect of Prdx1 on tumorigenesis. We used EC9706 cells to induce tumor formation in mice, using immune-deficient nude mice as a transplant carrier to avoid xenogeneic exclusion [35, 36]. Negative control EC9706 cells in logarithmic growth phase and EC9706 cells with inhibited Prdx1 levels were prepared as cell suspensions and injected subcutaneously into the right axillary of nude mice. Live imaging was used to detect the tumor formation capacity, and the radiant efficiency and distribution of luciferase luminescence were used as indicators of tumor formation ability and tumor invasive capacity, respectively. The results showed that the average radiant efficiency of luciferase luminescence was decreased 1.25-fold times when Prdx1 was inhibited, relative to the level in the negative control group. Additionally, the cells in which Prdx1 was inhibited also showed a decrease in the distribution of luciferase luminescence (Fig. 6a, b). These results suggest that Prdx1 inhibition significantly decreased both tumor formation ability and tumor invasive capacity. The mice tumor weight and volume also decreased significantly after inhibition of Prdx1 (Fig. 6c-e). We also removed the mice tumors and used immunofluorescence to detect the cilium marker protein Acetyl-α-tubulin. The results showed that the formation of tumor cell cilia was significantly increased following Prdx1 inhibition compared with this process in the control group (Fig. 6f, g). The above result suggests that Prdx1 can promote the occurrence and progression of esophageal squamous carcinoma by inducing cilia disaggregation. The inhibition of Prdx1 expression promoted cilia regeneration and inhibited the invasion of tumor cells.

### Decreased Prdx1 down-regulated the cilium disaggregation signal axis NEDD9-Aurora A-HDAC6 in tumor tissue

**Fig. 7:**
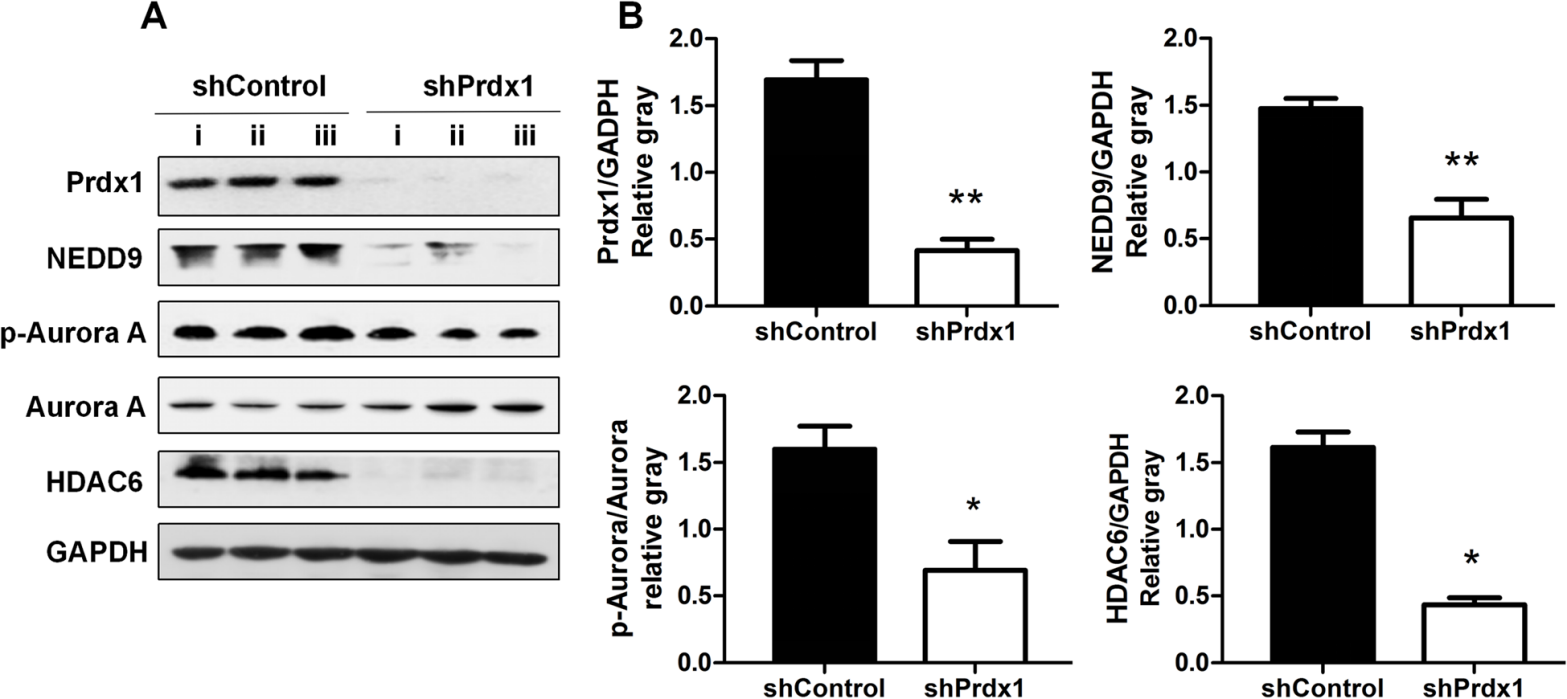
Decreased Prdx1 down-regulated the cilium disaggregation signal axis NEDD9-Aurora A-HDAC6 in tumor tissue. **a** Western-blot was used to detect changes of Prdx1, NEDD9, p-Aurora A, Aurora A, HDAC6, and GAPDH in tumor tissue of each group. **b** Analysis of western blot signals using Image J. GAPDH was used as a loading control. The relative protein levels are indicated as bars. Full-length blots/gels are presented in Supplementary Fig. X. All experiments were performed in triplicate and results are expressed as mean ± SEM. *P < 0.05, **P < 0.01, ***P < 0.001

We further analyzed the intracellular proteins of animal tumors in nude mice. The expression of Prdx1 was decreased in the shPrdx1 group compared with the level in the negative control group. Additionally, the expression levels of intracellular NEDD9, p-Aurora A, and HDAC6 were also significantly decreased in the shPrdx1 group (Fig. 7). This was consistent with the above cellular assay results, and further indicated that the action of Prdx1 to promote carcinoma cilium disaggregation and tumor invasion in esophageal squamous cells was achieved by modulation of the NEDD9-Aurora A-HDAC6 signal axis.

## Discussion

Prdx1 is a member of the peroxide oxidation-reduction enzyme family, and its abnormal expression is closely associated with multiple types of tumors [37–39]. Recent studies indicated that the high expression of Prdx1 can promote the occurrence and progression of esophageal carcinoma and that inhibition of Prdx1 expression can promote radiation and chemotherapy sensitivity for esophageal carcinoma treatment [40, 41]. We previously found that Prdx1 can promote cilia disaggregation in ESCC and strengthen the invasion capacity of tumor cells. Additionally, the inhibition of Prdx1 expression can promote cilium regeneration and down-regulate the activity of the cilium disaggregation marker protein Aurora A [3, 10]. Here, inhibition of the expression of Prdx1 in ESCC cells significantly up-regulated genes related to cilium regeneration and down-regulated genes related to cilium disaggregation. Cell and animal experiments demonstrated that Prdx1 plays a role in regulation of the critical NEDD9-Aurora A-HDAC6 signal axis of cilium disaggregation and influences the tumor formation and invasion capacity of ESCC cells. These results showed that Prdx1 was a critical regulator of cilium disaggregation in ESCC, making it an important target for treatment.

Inhibiting Prdx1 expression with the shPrdx1 lentivirus promoted cilia formation in EC9706 cells and inhibited the invasion capacity of the tumor cells. In nude mice, the tumor formation capacity, the tumor volume, and tumor weight were significantly decreased after inhibition of Prdx1. Thus, Prdx1 can promote the occurrence and progression of ESCC by inducing cilia disaggregation. Our results are consistent with other findings that inhibition of Prdx1 can inhibit the occurrence, invasion, and metastasis of tumors [42, 43]. However, multiple mechanisms have been proposed to explain Prdx1’s effects on cilia depolymerization. Chae Soomin et al. [44] found that Prdx1 knockdown markedly inhibited proximal tubule formation in the pronephros during embryogenesis, significantly increased the cellular levels of reactive oxygen species (ROS), and impaired primary cilia formation, findings that do not agree with our observations. The difference in activities may reflect different roles of Prdx1 in cilia formation for different cellular components and different disease conditions. Additional experiments are warranted to expand these experiments to characterize the effect of Prdx1 on cilia.

Previous research showed that the inhibition of Prdx1 can down-regulate the expression of Aurora A, a key protein in cilium disaggregation [3, 10]. Recent research has also shown that the NEDD9-Aurora A-HDAC6 signal axis plays an important role in this process [45, 46]. Based on our results, we propose that the action of Prdx1 on cilium disaggregation may be achieved through modulation of the NEDD9-Aurora A-HDAC6 signal axis. Our results showed significantly decreased expression of NEDD9, Aurora A, and HDAC6 when Prdx1 was inhibited with the shPrdx1 lentivirus. The expression of NEDD9, Aurora A, and HDAC6 also increased significantly when Prdx1 was over-expressed under the action of the OE-Prdx1 lentivirus. Stimulating the ESCC cells with the Aurora A inhibitor Tripolin A reversed the effect of the over-expressed Prdx1 on p-Aurora A and HDAC6 proteins, and decreased p-Aurora A enhancement of cilium regeneration and inhibited the invasion of tumor cells. Therefore, the effects of Prdx1 to strengthen the cilium disaggregation and invasion capacity of ESCC cells were mediated through regulation of the NEDD9-Aurora A-HDAC6 signal axis. Both Prdx1 and the NEDD9-Aurora A-HDAC6 signal axis play important roles in the occurrence and progression of ESCC, making both factors potential targets for treatment.

Several studies have showed that Prdx1 promotes tumor occurrence mainly by adjusting the levels of ROS, TGF-β1, mTOR/p70S6K, and ROS/ERK/cyclin D1 [47–49]. However, the effect and mechanism of Prdx1 on esophageal squamous cell carcinoma still need to be further explored. Here, we demonstrated that the NEDD9-Aurora A-HDAC6 signal pathway plays an important role in cilium disaggregation and tumor invasion. The results from the animal and cell culture experiments demonstrated that the signal axis mediates the role of Prdx1 to promote the occurrence and progression of cilium disaggregation and ESCC. There may be additional molecular mechanisms through which Prdx1 can influence the occurrence and progression of ESCC. Additionally, the applicability of the observed effects of Prdx1 on the NEDD9-Aurora A-HDAC6 signal axis to other tumor aspects requires future study.

## Conclusion

Our results suggest that Prdx1 is a novel regulator of primary cilia formation in ESCC cells.

## Supplementary information

**Additional file 1: Table S1.** Changes in the expression of different genes after Prdx1 inhibition in EC9706 cells. Intracellular differentially expressed genes after Prdx1 inhibition are presented in Table S1. The screening standard for a significant difference in gene expression was a value of |FC| greater than 1.3 and a *P*-value less than 0.05. Chip probe number and the FC information of the probe (Fold Change, the ratio of the expression of the treatment group to the control group) are listed.

**Additional file 2: Table S2.** Differentially expressed genes in disease and cell functional classifications after Prdx1 inhibition in EC9706 cells. All diseases and cell functions were sorted using -Log (*P*-value) and are ranked from high to low according to the value of -log (*p*-value), indicating the relationships between differentially expressed genes and the activation or inhibition of the disease and cell function. (XLS 8367 kb)

**Additional file 3: Table S3.** The occurrence of differentially expressed genes in classical signaling pathways. We studied the occurrence of significantly differentiated genes in classical signaling pathways based on data from public databases and the results of gene detection. We provide an enrichment analysis of the list of differentially expressed genes and the set of genes in the various classical pathways. We calculated the significance of *P*-value to determine which pathways of differentially expressed genes are enriched, as indicated in the classical pathway analysis column. A Z-score is given to indicate which pathways are activated or suppressed, based on the up/down regulation of genes according to chip analysis and the literature. (XLS 759 kb)

**Additional file 4: Figure S1.** Differentially expressed genes in classical signaling pathways. The signal pathway histogram shows the enrichment of differentially expressed genes in classical signaling pathways. All signal pathways are sorted using -Log (*P*-value). A larger -Log (*P*-value) indicated a more significant the enrichment of the pathway in the experimental results, and suggests a greater contribution of the pathway under that experimental condition. The orange marked signal pathways in the picture represents Z-score > 0, while the blue marked signal pathway indicates Z-score < 0. The Z-score shows the extent of activation or inhibition of the pathway under the experimental condition. The Z-score > 2 represents significant activation of the pathway, and the Z-score < − 2 represents significant inhibition of the pathway. The ratio represents the ratio of the number of differentially expressed genes to all the genes in the signal pathway.

**Additional file 5: Figure S2.** Detection of mRNA levels of downstream factors after Prdx1 inhibition. **A-D** Quantitative RT-PCR analysis was used to determine the relative mRNA level of Prdx1, FGFR1, IGFR1, and ABI2. **E-G** Quantitative RT-PCR analysis was used to determine the relative mRNA level of NEDD9, Aurora A, and HDAC6. GAPDH was used as a loading control. All experiments were performed in triplicate and the results are expressed as mean ± SEM. *P < 0.05, **P < 0.01, ***P < 0.001.

**Additional file 6: Figure X.** The original images of the blot and gel figures.

## Data Availability

All data generated or analysed during this study are included in this published article [and its supplementary information files].

## Abbreviations

Prdx1: Peroxiredoxin 1
ESCC: esophageal squamous cell carcinoma
HEF1: human enhancer of filamentation 1
HDAC6: Histone Deacetylase 6
