# Supplementary figures and images for: Prdx1 promotes the loss of primary cilia in esophageal squamous cell carcinoma
Source: BMC Cancer. 2020 May 1;20:372. doi: 10.1186/s12885-020-06898-y (PMC7195802; doi:10.1186/s12885-020-06898-y)

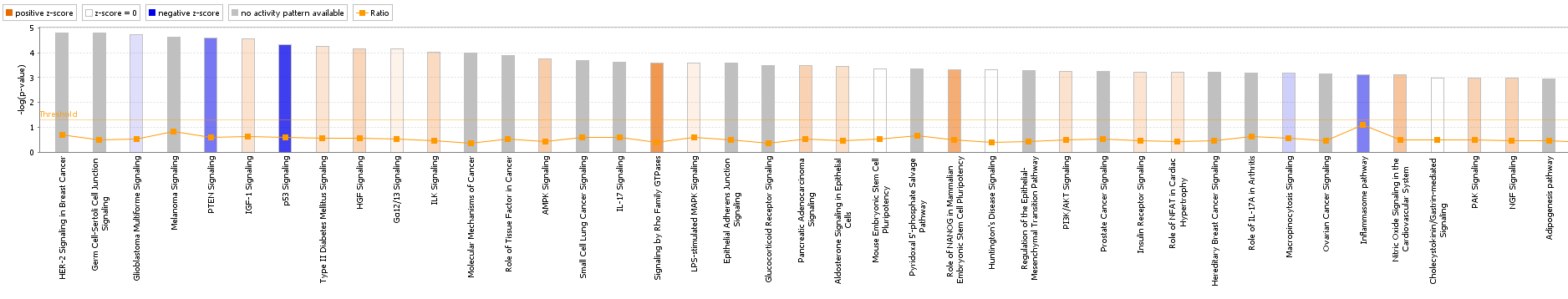

Supplement: Supplementary file 4 — Additional file 4: Figure S1. Differentially expressed genes in classical signaling pathways. The signal pathway histogram shows the enrichment of differentially expressed genes in classical signaling pathways. All signal pathways are sorted using -Log (P-value). A larger -Log (P-value) indicated a more significant the enrichment of the pathway in the experimental results, and suggests a greater contribution of the pathway under that experimental condition. The orange marked signal pathways in the picture represents Z-score > 0, while the blue marked signal pathway indicates Z-score < 0. The Z-score shows the extent of activation or inhibition of the pathway under the experimental condition. The Z-score > 2 represents significant activation of the pathway, and the Z-score < − 2 represents significant inhibition of the pathway. The ratio represents the ratio of the number of differentially expressed genes to all the genes in the signal pathway. [file 12885_2020_6898_MOESM4_ESM.tif]

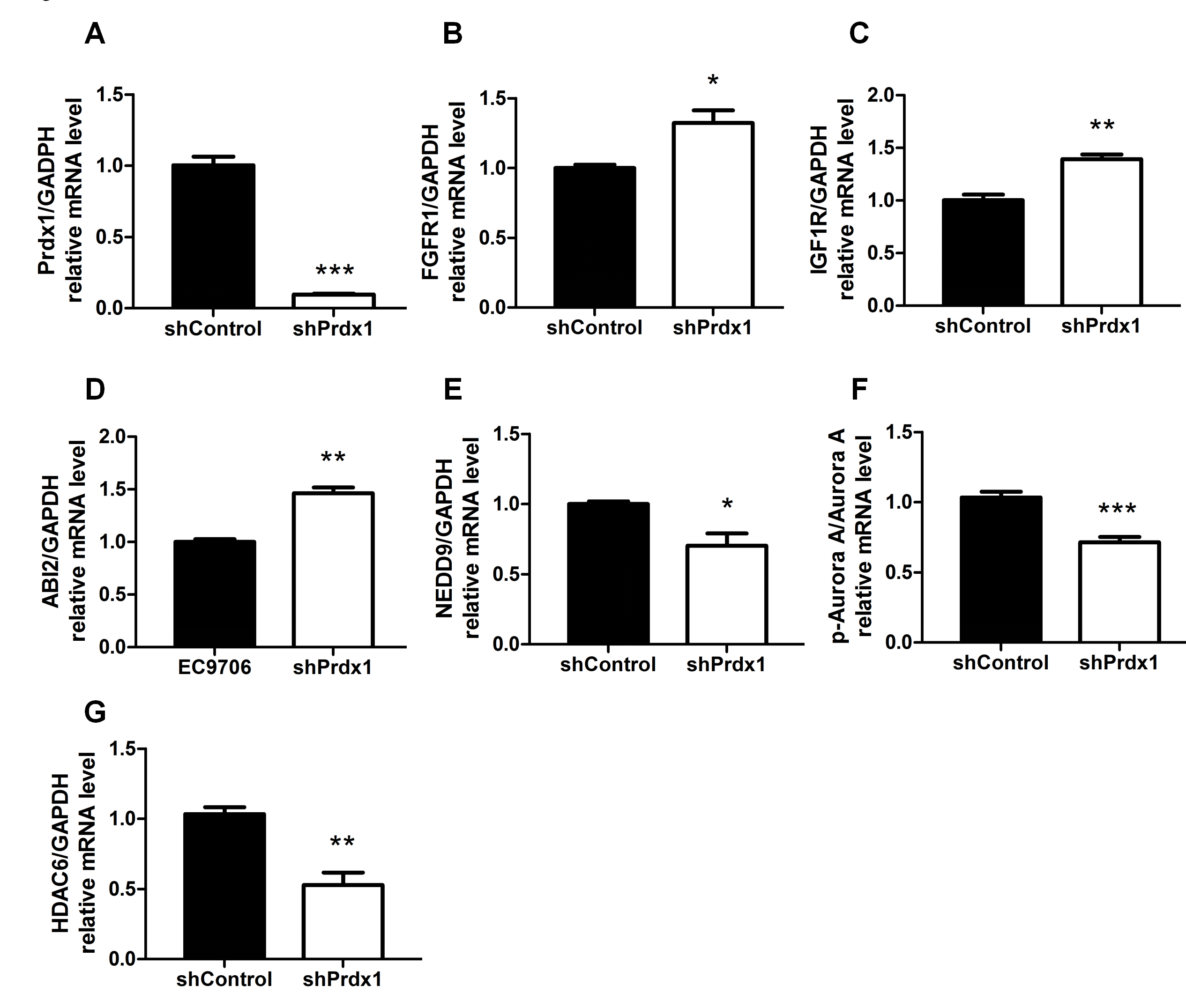

Supplement: Supplementary file 5 — Additional file 5: Figure S2. Detection of mRNA levels of downstream factors after Prdx1 inhibition. A-D Quantitative RT-PCR analysis was used to determine the relative mRNA level of Prdx1, FGFR1, IGFR1, and ABI2. E-G Quantitative RT-PCR analysis was used to determine the relative mRNA level of NEDD9, Aurora A, and HDAC6. GAPDH was used as a loading control. All experiments were performed in triplicate and the results are expressed as mean ± SEM. *P < 0.05, **P < 0.01, ***P < 0.001. [file 12885_2020_6898_MOESM5_ESM.tif]
