# Supplementary material for: Prdx1 promotes the loss of primary cilia in esophageal squamous cell carcinoma
Source: BMC Cancer. 2020 May 1;20:372. doi: 10.1186/s12885-020-06898-y (PMC7195802; doi:10.1186/s12885-020-06898-y)
Supplement: Supplementary file 6 — Additional file 6: Figure X. The original images of the blot and gel figures. [file 12885_2020_6898_MOESM6_ESM.docx]

Figure 2

OE-Control

shPrdx1

shControl

OE-Prdx1


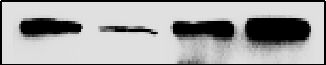
 Prdx1 22 KD


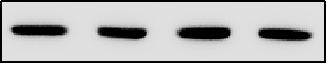
 GAPDH 37 KD

Figure 3 A

shPrdx1

shControl

shPrdx1

shControl

EC9706

EC9706


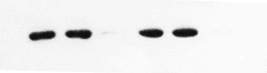
Prdx1 22 KD


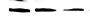
 NEDD9 115 KD


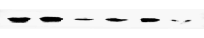
 p-Aurora A 48 KD


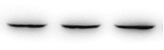
 Aurora A 46 KD


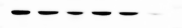
HDAC6 131 KD


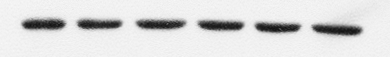
GAPDH 37 KD

Figure 3 C

OE-Prdx1

EC9706

OE-Control

OE-Prdx1

OE-Control

EC9706


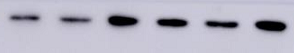
 Prdx1 22 KD


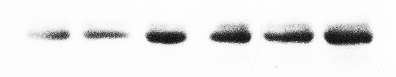
NEDD9 115 KD


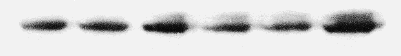
p-Aurora A 48 KD


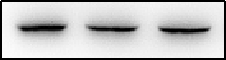
 Aurora A 46 KD


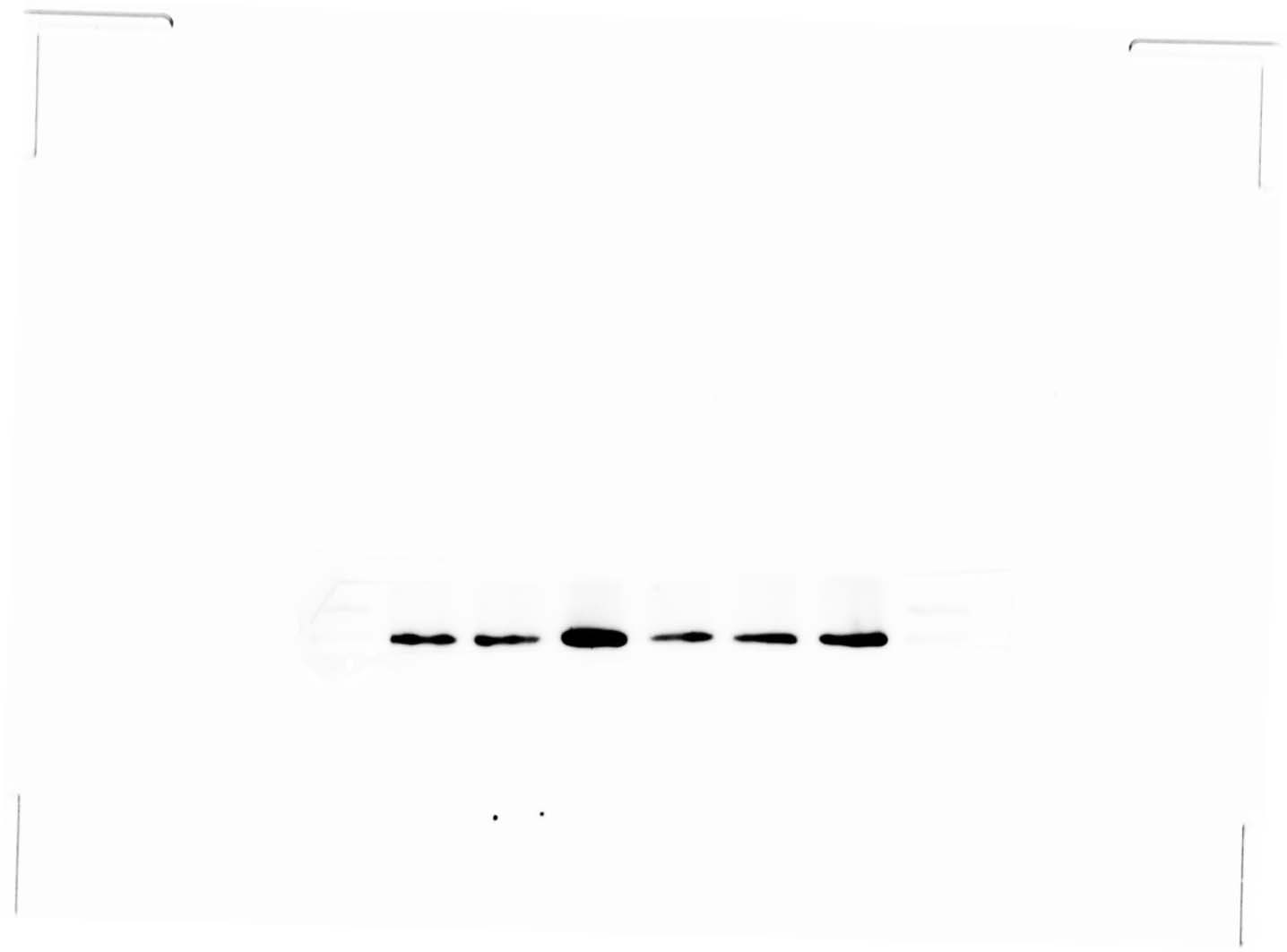
 HDAC6 131 KD


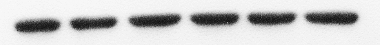
 GAPDH 37 KD

Figure 4 A

Tripolin A：

0 1.5 4.5 7.5 10.5 (µmol/L)


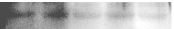
 p-Aurora A 48 KD


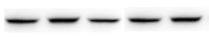
 Aurora A 46 KD


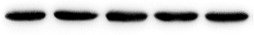
 GAPDH 37 KD

Figure 4 C

Tripolin A (4.5 µmol/L)

0 2 4 8 12 (h)


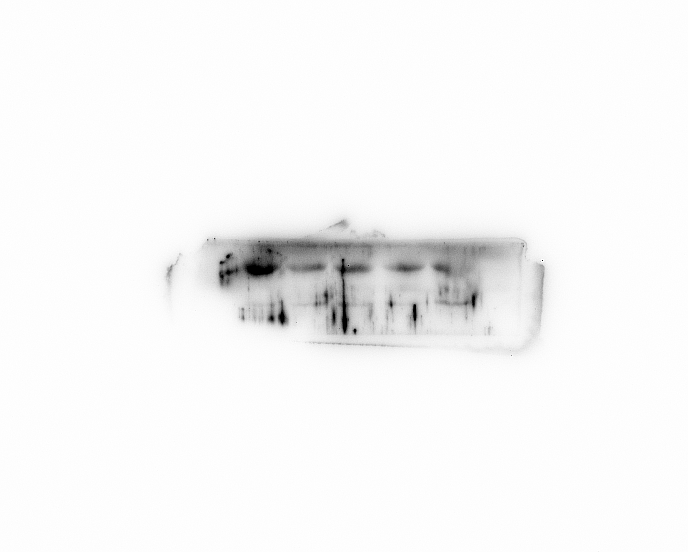
p-Aurora A 48 KD


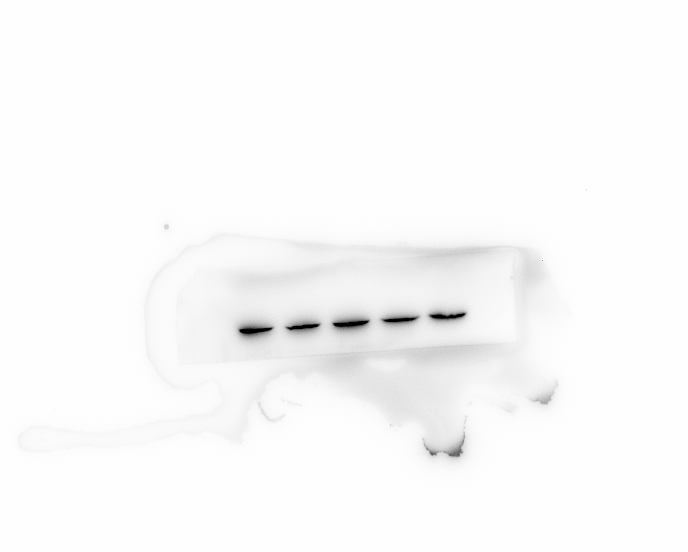
 Aurora A 46 KD


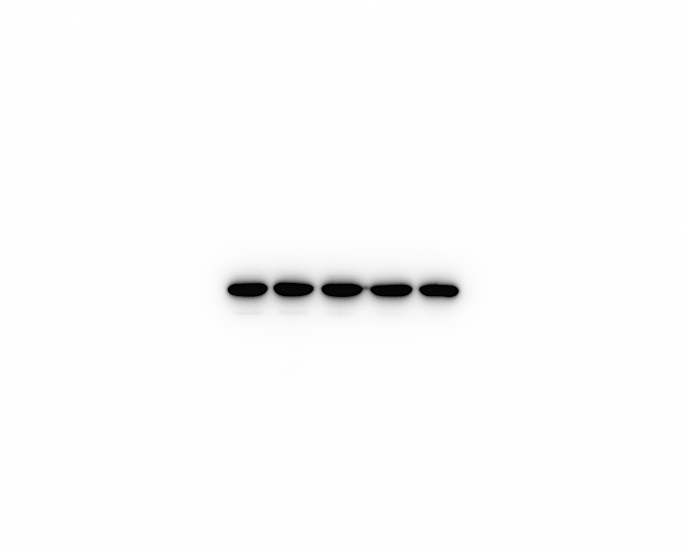
GAPDH 37 KD

Figure 4 E

EC9706+Tripolin A

EC9706


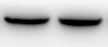
 NEDD9 115 KD


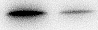
 p-Aurora A 48 KD


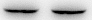
 Aurora A 46 KD


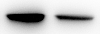
 HDAC6 131 KD


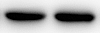
 GAPDH 37 KD

Figure 4 G

OE-Prdx1+Tripolin A

OE-Prdx1

OE-Control


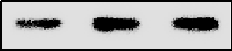
 NEDD9 115 KD


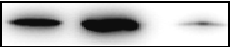
 p-Aurora A 48 KD


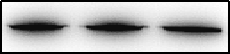
 Aurora A 46 KD


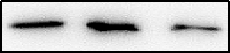
 HDAC6 131 KD


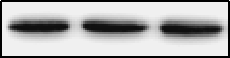
 GAPDH 37 KD

Figure 7 A

shControl shPrdx1


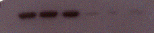
 Prdx1 22 KD


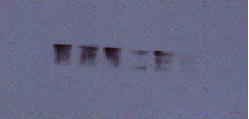
 NEDD9 115 KD


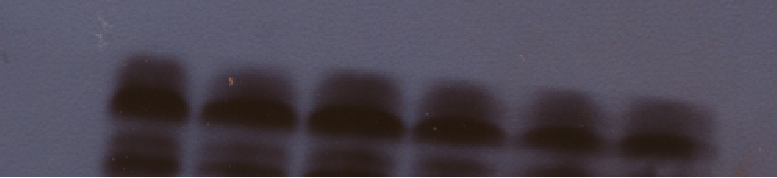
 p-Aurora A 48 KD


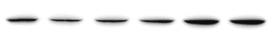
 Aurora A 46 KD


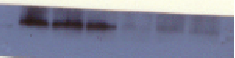
 HDAC6 131 KD


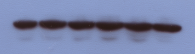
 GAPDH 37 KD
